# Clinical assessors’ working conceptualisations of undergraduate consultation skills: a framework analysis of how assessors make expert judgements in practice

**DOI:** 10.1007/s10459-020-09960-3

**Published:** 2020-01-29

**Authors:** Catherine Hyde, Sarah Yardley, Janet Lefroy, Simon Gay, Robert K. McKinley

**Affiliations:** 1 School of Medicine, Keele University, Keele, Staffordshire, ST5 5BG UK; 2 Palliative Care Service, Central and North West London NHS Foundation Trust, St Pancras Hospital, 5th Floor South Wing, 4 St. Pancras Way, London, NW1 0PE UK; 3 University of Leicester School of Medicine, Leicester, UK

**Keywords:** Clinical skills, Education, medical, undergraduate, Education, professional, Judgement, OSCE, Professional judgment, Qualitative research, Rater cognition, Rater judgments, Theory of expertise

## Abstract

Undergraduate clinical assessors make expert, multifaceted judgements of consultation skills in concert with medical school OSCE grading rubrics. Assessors are not cognitive machines: their judgements are made in the light of prior experience and social interactions with students. It is important to understand assessors’ working conceptualisations of consultation skills and whether they could be used to develop assessment tools for undergraduate assessment. To identify any working conceptualisations that assessors use while assessing undergraduate medical students’ consultation skills and develop assessment tools based on assessors’ working conceptualisations and natural language for undergraduate consultation skills. In semi-structured interviews, 12 experienced assessors from a UK medical school populated a blank assessment scale with personally meaningful descriptors while describing how they made judgements of students’ consultation skills (at exit standard). A two-step iterative thematic framework analysis was performed drawing on constructionism and interactionism. Five domains were found within working conceptualisations of consultation skills: Application of knowledge; Manner with patients; Getting it done; Safety; and Overall impression. Three mechanisms of judgement about student behaviour were identified: observations, inferences and feelings. Assessment tools drawing on participants’ conceptualisations and natural language were generated, including ‘grade descriptors’ for common conceptualisations in each domain by mechanism of judgement and matched to grading rubrics of Fail, Borderline, Pass, Very good. Utilising working conceptualisations to develop assessment tools is feasible and potentially useful. Work is needed to test impact on assessment quality.

## Introduction

Consultation skills such as obtaining a medical history and performing a physical examination are core elements of undergraduate medical education (General Medical Council 2011; Novack et al. 1993; Sankarapandian et al. 2014; Stillman et al. 1997; Townsend et al. 2001) but their assessment is challenging (Schuwirth and van der Vleuten 2006). OSCEs have been found to be feasible (Patricio 2012) and can facilitate reliable assessment of undergraduate consultation skills (Patricio 2012). As OSCEs have come to ‘dominate’ skills assessment (Cömert et al. 2016; Norman 2002), there is increasing interest in ways of improving the quality of high stakes assessment, with particular focus on the determinants of reliability (Van der Vleuten 1996) which is often unsatisfactory (Brannick et al. 2011).

It is challenging to increase the reliability of assessor judgements because of the relational nature of assessor judgements (Gingerich et al. 2018; Hope and Cameron 2015; Yeates et al. 2012, 2015) and the minimal impact of training on inter-rater reliability (Cook et al. 2009; Holmboe et al. 2004). There is little published research on undergraduate assessor cognition. A recent systematic review (Lee et al. 2017) identified three studies of undergraduate assessment. In two, undergraduate workplace based performances were assessed by assessors recruited on the basis of their expertise in assessing postgraduate general practice trainees (Govaerts et al. 2011, 2013) and the third examined the product (scores) of assessment rather than the cognitive process (Rogausch et al. 2015). While assessor judgements are highly context dependent (Gingerich et al. 2018; Govaerts et al. 2011; Hope and Cameron 2015; Yeates et al. 2012, 2015) recent research about assessor judgements in post graduate work based assessment may inform our thinking about undergraduate OSCE assessment. This work has drawn on social and cognitive psychology to understand the processes of how humans make judgments (Eva 2018; Gingerich et al. 2014; Govaerts et al. 2013; Yeates et al. 2013, 2015). Variability in assessor judgements can be understood as assessors applying ‘meaningfully idiosyncratic’ (Gingerich et al. 2014) working conceptualisations. For the purpose of this paper we define a working conceptualisation as a meaningful idea which underpins a domain of judgement generated through interaction between assessor and student. ‘Translating’ judgments into scales is key to the rating process (Gauthier et al. 2016). Reduced assessor reliability may be partially explained by poor alignment between assessors ‘meaningfully idiosyncratic’ (Gingerich et al. 2014) working conceptualisations and the ‘external’ rubric with which they are asked to communicate their judgement, thus introducing error and variability (Gingerich et al. 2011). It is noteworthy that, in postgraduate assessment, assessments of doctors in training by assessors using scales which reflect the assessors’ own working conceptualisations (construct aligned scales) are more reliable (Crossley et al. 2011). It is possible therefore, that undergraduate OSCE assessments would be more reliable if tools aligned to assessors’ working conceptualisations were used.

Multiple tools are used to assess different aspects of undergraduate consultation skills, many of which are specific to individual medical schools (Setyonugroho et al. 2015). While some are theoretically informed (Humphris and Kaney 2001; Huntley et al. 2012) and others based on national criteria (Kaul et al. 2012) or consensus based models such as the Calgary Cambridge model and its derivatives (Lefroy et al. 2011; Silverman et al. 2011), none were developed to align with assessors’ working conceptualisations. Although Govaerts et al. (2013) have described clinician assessors’ internal (or working) assessment ‘dimensions’ in the postgraduate context and Gingerich et al. (2018) described ‘clusters’ of individual assessor judgement, it is unknown whether undergraduate assessors hold such working conceptualisations nor if they form clusters which may be useful in assessment tools. For example, clinical assessors who are expert and experienced in their field may be less equipped to translate their working conceptualisations of consultation skills to the undergraduate exit standard which is remote from their own practice.

This research aims to take the first steps in determining whether undergraduate assessors hold such working conceptualisations and if they form clusters which may be useful in assessment tools by:

- Identifying any working conceptualisations that assessors use while assessing undergraduate medical students’ consultation skills.
- Developing assessment tools based on assessors working conceptualisations and natural language for undergraduate consultation skills.

## Methods

### Theoretical and epistemological orientation

Our conceptual orientation is towards the principles of constructionism and interactionism: people construct meaning through interpretation. Constructionism is the view that “all knowledge, and therefore all meaningful reality as such, is contingent upon human practices, being constructed in and out of interaction between human beings and their world, and developed and transmitted within an essentially social context” (Crotty 1998 p. 42). Unlike constructivism (which focuses on the individual mind) constructionism emphases more strongly how we are influenced by culture and interactions—and hence is considered by many social scientists to sit relatively closely on a spectrum of theoretical worldviews to interactionism (Denzin 2001). Working conceptualisations ‘may influence observations and judgements about other people by providing frames-of-reference or sets that make perceivers look for certain kinds of interpersonal information and interpret this information according to their own conceptualisations’ (Borman 1987). ‘Working conceptualisation’ in this specific context is a meaningful idea which underpins a domain of judgement generated through interaction between assessor and student. Meaning making is an iterative process developed through each person’s presentation of themselves and interpretations generated through their interaction mediated by the environment and situation (Blumer 1969; Crotty 1998; Goffman 1967). While recognising the differing terminology in this field, ‘working conceptualisation’ is used intentionally as it best reflects our orientation.

### Context

The study was performed at a UK undergraduate medical school where teaching and assessment of consultation skills are underpinned by an assessment tool used in both formative work-based assessment (WBA) and summative objective structured clinical examinations (OSCEs) (Lefroy et al. 2011). Assessors attend training sessions prior to using the tool as is accepted good assessment practice (General Medical Council 2011; Khan et al. 2013). Research ethics approval was given by the School’s Ethics Committee (ref date 16/08/12).

### Recruitment and participation

All undergraduate clinical assessors with at least 2 years’ experience of making high stakes assessments [a previously used standard (Ginsburg et al. 2010)] for a single UK medical school were invited by email to participate (n = 64). Responding assessors were purposively sampled using length of assessment experience as a proxy for assessment expertise (Govaerts et al. 2013). Further sampling of assessors sought variation in age, gender and clinical speciality (Patton 2002). Recruitment continued until theoretical saturation of key conceptualisations occurred (n = 12).

### Data collection

Our aim was to encourage assessors to access their own internal working conceptualisation of undergraduate consultation competence by asking assessors to populate an unmarked line (a blank scale) with their own descriptors of differing levels of performance. During five pilot interviews (X, n = 2; Y, n = 3), we determined that some assessors could not work with a blank scale so we developed a scale with reference points of ‘Clear pass’, ‘Borderline’ and ‘Clear fail’ (“Appendix 1”) to enable discussion if assessors could not successfully populate the entirely ‘blank’ scale. We also developed a semi-structured interview topic guide (“Appendix 2”). Pilot interviews were not included in the final analysis. CH and JL, who conducted all interviews, shared recordings of their first interviews to standardise and refine interview technique. Interviews were 40 to 60 min long, audio-recorded and contemporaneous field notes were kept.

In interview, participants were asked to describe the ‘global scale’ they used when judging a medical student to the standard of being ready to enter first year of training as a doctor [intern] (exit standard) by populating a scale with words and phrases. Participants were initially offered a completely blank scale. If they struggled, they were given the assessment scale developed in the pilots (“Appendix 1”). Participants were encouraged to elaborate their own definitions as they populated the scale. Each participant then described their working conceptualisations for two specific skill categories from the Medical School’s consultation skills assessment rubric (Lefroy et al. 2011). A matrix was used to ensure that all categories were considered by two or more participants during the study. These categories were: opening, history, examination, management, record keeping, case presentation, clinical reasoning, organisation, and building and maintaining the relationship (Lefroy et al. 2011). If participants’ overall judgement focused on any of these specific skill categories, that category was fully explored before revisiting the ‘overall’ scale to test for further potential conceptualisations. Novel categories and conceptualisations were discussed in detail when these emerged. In later interviews, relatively unexplored categories and emerging conceptualisations were presented to participants for discussion. Each participant was asked to complete two scales.

Participants were asked to describe specific student performances to illustrate their conceptualisations, drawing on cognitive interviewing (Willis 2005), critical incident (Choo et al. 2014) and think aloud techniques (Govaerts et al. 2013).

### Data analysis (see also“Appendix 3” for schedule of activities undertaken)

All authors contributed to the thematic analysis and critical review at each level of analysis (Braun and Clarke 2006). Framework analysis (Gale et al. 2013; Ritchie and Lewis 2003) used an initial coding framework developed from the original study protocol, research question and literature and was refined with the data. ‘Framework’ (Ritchie and Spencer 2002) is a qualitative analysis technique which involves researchers engaging their creative and critical conceptual skills to determine meaning and connections in data. The approach relies on ‘sifting, charting and sorting’ material into key issues and themes—also referred to as ‘indexing, charting and mapping/interpretation’—a process we achieved by creating word pictures, word summaries and grade descriptors from the data. In doing so we were creating a thematic framework drawing on a priori issues i.e. the research aims, objectives and questions, and emergent issues raised by our participants gradually organising these into analytical themes. We also followed recognised qualitative interpretative methods including constant comparison and returning to check raw data to ensure each level of interpretation drew on the raw data (Blumer 1969; Gale et al. 2013). At each stage we constantly compared back to raw data to ensure the analysis remained true to the data as a whole having familiarised ourselves with the data before starting the formal analytic process through listening to recordings and reviewing transcripts and participant annotations of scales. In this way we are confident that the final outcomes of the study represent the assessors’ collective natural language and meaning (Table 1).

**Table 1:** Glossary

Term	Definition
Descriptor	A significant word or phrase used to describe assessment dimension on an assessment scale
Domain	Identified area or facet of consultation skills e.g. Manner with patient
Exit standard	The standard of a medical student being ready to enter first year of training as a doctor [intern]
Grade descriptor	Description of each of the four grades, (fail, borderline, good, very good) synthesising all three types of judgements for each domain
Natural language	Words and phrases used by assessors themselves
Working conceptualisation	A meaningful idea which, in this specific context, underpins a domain of judgement generated through interaction between assessor and student. (See theoretical orientation in methods section for further detail)
Personally meaningful descriptors	Descriptors which individual participants assigned to judgements they made about students using their own words and phrases.
Types of judgement	3 ways participants made judgements of students: observations, inferences and feelings about the student’s behaviour
Word picture	Short description drawing on participants’ language (for each domain and type of judgement) which an assessor could use to place students on a scale
Word summary	Short summary of key conceptualisations (for each domain and type of judgement) drawing on ‘word pictures’ and raw data

Data tables are presented to help the reader follow this process (Tables 2, 3 and 4, “Appendices 3, 4, 6”).

**Table 2:** Skill domain ‘manner with patients’: illustrating how assessors’ raw data, with illustrating extracts were synthesised into ‘word pictures’ and ‘word summaries’ for each type of judgement: what the student does, what I infer, what this makes me feel. Note examples of data extracts are only shown for some grades due to space limitation

Judgement	Fail	Borderline	Pass	Very good
What the student does				
Example data extracts	Rushes in at the task and the patient is just another bit of the task, tense and uncomfortable, treating the patient carelessly or with no respect making the task the focus (10). If the patient is left undressed not explaining themselves, too much medical jargon, not at a level the person understands (8). Person who was the volunteer looked shocked and said “that was awful”; no engagement with the patient; weak; won’t stop talking; hurting the patient; not attend to the patient’s needs; No rapport (1). Unkind or rough to the patient (3)	Hesitant, not confident, do it in the wrong order/something in interviewee’s tone about the difference—not just the words but how they are said. Illustrated by how he says, ‘what’s wrong with you’ (12). Speak in language the patient doesn’t understand not adjusting examination technique or acknowledging pain; patient is surprised by action (9). Cursory attention to the patient at first, half and half attention to patient and task (10). Forget what question they asked, potentially ask the question a second time (1). Forget introduction, not aware of patient wincing (6)	Consent and talk them through it, minimum basic; some poor judgements but does not hurt the patient; Ability to pick up on cues (11). Nice to the patient; Handles interactions with relatives (3). Exploring options. Explain in useful chunks, suggests strategies and has empathy (7). Rapport with patient, not using medical jargon, opportunity to ask questions, open questions, exploratory questions, allowing patient to talk, not talking over them (4)	Display empathy, listen carefully and follow up the leads that patients give them, recognising if the patient has any understanding problems (e.g. hard of hearing), modify their voice (8). Take account of diet, social life; steer the conversation, guides their questions, listening; non-verbal contact; practical relatives management also (7). With examination, little bits of comment all the way through that show the patient that they’re being treated with respect. Thank them at the end (10)
Word picture	Patient reports concerns about student or seems upset. Doesn’t recognise cues about patients concerns, uses lots of jargon. Demonstrates judgmental behaviour. Does not direct the conversation, asking questions by rote or won’t stop talking. Not communicating at the right level, e.g. with children or so the patient needs to keep questioning. The student hurts the patient and does not recognise or manage this	Cursory attention to the patient, a very brief acknowledgement. Not adjusting consultation to the patient, i.e. continuing to speak in a quiet voice when the patient cannot here this. Ask questions a second time so the patient knows they are not listening. Discuss only clinical information. Focus mostly on the task. Demonstrated in lack of eye contact, disinterested tone, and phrasing e.g. ‘what’s wrong with you’	Introduces self to patient, explains consultation purpose to patient. Personable, nice to the patient. Involves the patient in the decision about management, gives opportunity for questions. Good consultation skills, uses open questions, exploratory questions, allowing patient to talk, not talking over them, explaining in chunks. Avoids medical jargon. Some errors but does not hurt or worry the patient	Demonstrates empathy, shows patient respect and is mindful of them. Takes care of the patient, checking the patient is comfortable during an examination, ensuring they are re-clothed afterwards. Recognises if the patient has any problems understanding and adjusting to this, modifying their voice. Prepares the patient for each part of the consultation. Steers the conversation. Uses non-verbal cues and contact. Manages relatives. Discusses social information
Word summary	Judgmental, ignores, hurts or upsets the patient	Cursory attention to, or acknowledgment of, or slow to adapt to patient’s needs	Rapport with and empathy for and comfortable with the patient	Empathic, prepares patient for what is next or might happen, adapts to the patient’s and family’s needs
What I infer				
Data extract	Impression doing things that would escalate badly in real life; wooden (4). Don’t understand the patient (3). Lacking confidence (7). Not quite comfortable, not impressive enough (10). Difficult for me to pass them (5)	Not had as much experience as they should, possibly upsetting a patient (9). The patient doesn’t feel listened to and starts to switch off from the doctor; having forgotten what’s already been said (6). Little conversation, conversing only the clinical bit, focusing on the task (10)	Human factor is missing, good level of conversation, the patient will go satisfied but not happy (11). Polite (9). Look comfortable talking to a patient (4)	Putting the patient at ease (11). Patient enjoys talking to them; the patient feels comfortable, as to what they’re doing next; looked like they’d done it before, the volunteer knew what was going to happen next (1). Showing respect; mindful of the patient; the right kind of approach (10)
Word picture	The student doesn’t understand the patient. The student has not talked to patients before, lacks confidence. The situation may escalate badly in real life. Focused on the task to the exclusion of the patient or treating the patient as part of the task. There is no rapport, the patient does not understand	Some patients may be upset by what the student has said. Students not used to talking with patients, has not been practicing consultations. Tick box consultation	Student is polite and can maintain a professional conversation. The patient will be satisfied with the consultation but not happy	Patient feels comfortable and at ease. The patient knows what’s going to happen next and will be happy with the consultation. Student has done this before
Word summary	Disregards or disrespects the patient, judgmental	Lacking in confidence, insufficient practice with patients	The patient is satisfied but not happy	Practiced, confident and competent respectful; patient enjoyed encounter
What this makes me feel				
Data extracts	Disrespects the patient, lack of care for the patient (10)	Able to pull self-up with interpersonal skills (9)	Just good enough (7). Involves patient in the decision (3). Kind to the patient, able to maintain a conversation (11). May do something the patient isn’t expecting (1)	Conveys a degree of reassurance that they know what they’re doing (1). Beginnings of patient doctor relationship (9)
Word picture	Sense that the student doesn’t care about the patient	Sense the student cares but needs to work on skills to be able to communicate with the patient. The student should be able to improve with support	Can maintain a professional conversation	Reassurance that student knows what they are doing. Able to be human and warm as well as professional. Creates the beginnings of a doctor-patient relationship
Word summary	Things could go wrong with patients	Seems to care but needs to learn how to communicate it. Can I trust the student not to upset patients?	The student may do something the patient isn’t expecting	I feel reassured (about skills to work with patients)

**Table 3:** ‘Word summaries’ for the three judgement types assessors made, shown for four specific skills domains identified (Knowledge, Manner with patients, Getting it done and Safety)

Type of judgement	Fail	Borderline	Pass	Very good
	*Knowledge*			
What the student did	Lack of comprehension and or working response	Incorrect approach but with evidence of potential to change	Essentially has the correct with suitable approach	Coherent synthesis of fluent consultation
What I inferred	Process focused, no synthesis	Lack of focus, notable omissions	Processing information, able to tailor approach in response	Good clinical judgement demonstrated, no longer process focused
What this made me feel	No practical understanding	Answers by accident not design	Inspires trust	Demonstrate capabilities, exceeding expectations
	*Manner with patients*			
What the student did	Judgemental, ignored, hurt or upset the patient	Cursory attention to, or acknowledgment of, or slow to adapt to patient’s needs	Rapport with, and empathy for, and comfortable with the patient	Empathic, prepares patient for what is next or might happen, adapts to the patient’s and family’s needs
What I inferred	Disregarded or disrespected the patient, judgemental	Lacking in confidence, insufficient practice with patients	Patient satisfied but not happy	Practiced, confident and competent respectful; patient enjoyed encounter
What this made me feel	Things could go wrong with patients	Seems to care but needs to learn how to communicate it. Can I trust the student not to upset patients?	May do something the patient isn’t expecting	Reassured (about skills to work with patients)
	*Getting it done*			
What the student did	The task is incompletely done because of patchy, slow, technique or misdirected focus	Just about did the task. Some bits wrong or missing, disorganised	Hesitant but thoughtful. Not very graceful. Gets the task done but messily	The task is completed and flows smoothly with a systematic observant approach
What I inferred	Incompetent. Clearly didn’t have a clue; focus is wrong	They don’t really know what they’re doing	They look like they know what they’re doing	Confident and know exactly what they are doing
What this made me feel	Couldn’t trust them with this task in real life	I’m slightly worried that they are likely to miss things out	Gets the task done without impressing me hugely	Makes you feel that this person really knows what they’re doing
	*Safety*			
What the student did	Actions which cause harm or compromise safety	Mistakes made but overall not dangerous	Responds to errors in a safe manner	Safe, correctly focused
What I inferred	Unsafe, has no insight	Safety possibly compromised by anxiety	Some awareness of potential harm or dangers	Safe, fluent
What this made me feel	Cannot be trusted	Remediable

**Table 4:** ‘Grade descriptors’ for each of the five domains of consultation skills

Domain	Grade descriptors			
	Fail	Borderline	Pass	Very good
Knowledge	Appears to follow a routine without any understanding with evidence of one or more of: comprehension, working response, synthesis or practical understanding	Mixed evidence between Fail and Pass descriptors. Some sense of potential to improve	Evidence of tailored approach with analysis of situation as consultation progresses. Inspires confidence in ability to provide immediate care	Synthesis of fluent consultation. Exceeding expectations
Manner with patients	Judgmental; likely to ignore, hurt or upset patients	Seems to care but needs to learn how to communicate it. Can I trust the student not to upset patients?	Empathic, unlikely to upset patients	Anticipatory empathy, anticipates and explains problems before they arise
Getting it done	Nowhere near getting it done	Kind of got it done	Got the task done reasonably well	Task done well. Observant, slick and systematic
Safety	Attitude of conscious or unconscious incompetence	Mistakes but overall not dangerous, can improve	Awareness and insight into own abilities and able to rectify mistakes	Safe and fluent
Overall impression	(I have) Concerns about the student having contact with patients or progressing further in the course	Struggling to manage emotions or accept responsibility for patient care. Minor issues that student will work on and can be supported to improve	Performs as taught. Beginning to think and act like a doctor	Performs like a doctor. Conscientious, compassionate, in control of themselves and the situation

### Primary analysis within and across individual interviews

The audio-recording and scales from each interview were analysed by the respective interviewer and another team member. The interviewer listened to the interview, transcribing data extracts and commenting on their relation to skill categories and emerging working conceptualisations. This process was recorded in a coding table (indexing) developed during the pilot interviews so that all research team members could review the evolving analysis (“Appendix 4”). Words and phrases used by participants to describe the ‘fail’, ‘borderline’ and ‘pass’ grades were recorded. A ‘very good’ column was added when it became apparent that participants’ working conceptualisations were distinguishing the passing student from the high performing student. The second researcher then reviewed the recording, critiqued the interviewer’s interpretation, added additional data extracts and explored alternative interpretations. The pairing discussed their analysis and any differences in interpretation to reach consensus. The emerging coding structure (framework development) was discussed at research team round-table meetings when pairs presented their findings. A quality check was performed by a third reviewer for each pairing and each interviewer worked with all team members during the analysis. The analysis iteratively informed content of subsequent interviews.

After 12 interviews there was consensus that no new domains or judgement mechanisms were emerging, and the final interviews had added little. Data from all interviews were combined in table format and all researchers re-analysed the interviews seeking data extracts which confirmed or challenged provisional findings of domains and judgement mechanisms (charting). A second researcher reviewed each domain table critically for alternative explanations.

### Secondary analysis of data across domains and judgement mechanisms

Data extracts were integrated into short descriptions drawing on participants’ natural language and conceptualisations to create ‘word pictures’ (stage 1 mapping and interpretation) which could be used to place students on a scale. These ‘word pictures’ were summarised drawing on the raw data to identify key conceptualisations in the form of ‘word summaries’ (stage 2 mapping and interpretation). These ‘word summaries’ permitted a global overview of the data and were discussed and critiqued at a round-table meeting. The terms ‘word picture’ and ‘word summary’ evolved during conception of the study and analysis of the data. In the final stage (stage 3 interpretation) ‘grade descriptors’ were developed to synthesise all three judgement mechanisms for each of the four grades for each domain. These final ‘grade descriptors’ drew on the ‘word pictures’ and ‘word summaries’, as well as the raw data and participants’ comments about how they graded students. ‘Grade descriptors’ were reviewed and critiqued by a second researcher, then discussed at a round-table meeting. In the case of ‘overall impression’ a second round of reviewing and critique was performed to capture this domain’s complexity in the ‘grade descriptors’. At each stage of the analysis we checked back to the previous stage and the original data to ensure consistency with the language used by assessors. This ensured the natural language was used to create the products of our analysis and drew on it in generating the descriptors. This process of developing ‘grade descriptors’ is further described in “Appendix 3”.

## Results

12 (7 female) experienced clinician assessors were recruited from 11 different clinical specialties. Each had assessed students in at least 10 OSCEs. They were 39 to 56 years old, had 4 to 29 year’s teaching experience and 7 had experience in completing formal workplace-based assessments on students. As well as being undergraduate assessors, all participants had other postgraduate teaching or assessment experience (“Appendix 5”). Of the 24 scales populated by the 12 participants, 5 were scales pre-populated with reference points including one scale annotated by the participant (“Appendix 1”).

Key findings of the research are described below: participants’ three judgement mechanisms and three examples of the five cross-cutting skill domains are presented first. Assessors’ working conceptualisations identified in the iterative analysis are highlighted within the descriptions of the domains and illustrated by the ‘word summaries’ (Table 3). We found ‘word pictures’, ‘word summaries and ‘grade descriptors’ had potential for development into assessment tools, within assessment scales or an assessment matrix. Examples of ‘word pictures’ are described, which could be used to place students on a scale, and ‘word summaries’ which identify key conceptualisations alongside the domains with further examples in Table 2 and “Appendix 6”. Exemplar ‘grade descriptors’ are also presented and fully detailed in Table 4.

### Judgement mechanisms

Assessors used three judgement mechanisms: observations of students’ behaviour, inferences and feelings about the student’s behaviour (Box 1). Within application of their working conceptualisations, participants often discussed one mechanism of judgement only for specific elements of their assessment and were not always able to describe what student behaviour had generated an inference or feeling when these mechanisms were drawn on. However, most drew on all three judgement mechanisms across the working conceptualisations applied by assessors at different times for different elements of assessment, for example an assessor could make an observation about one domain early in the consultation, an inference about another later and have a feeling about the first late in the consultation. This highlights the complexity of applied judgement drawing on working conceptualisations, confirming that these experienced and trained assessors do not mechanically apply rubrics.

**Box 1:** The three judgement types as used by Assessor 1

Assessor 1’s comments	Type of judgement
“*Clearly didn’t know what he was doing*. Felt for pulses in some interesting places and then told me he could feel a bounding pulse when I knew he couldn’t feel a pulse in that part of the body. Couldn’t even find the femoral pulse on the simulator- didn’t know where to find the femoral pulse on the simulator…. I didn’t like the fact that he told me he could feel a pulse when he couldn’t possibly be feeling pulses, *which meant that he was lying*. Making up physical signs, making out you can find something when you can’t … no way I can trust that person to be my house officer [intern], to know that anything he’s found or says he’s found is true. And the complete lack of any knowledge of where nearly all the pulses were. *That enormous gap in knowledge*	*Inference* Observation Feeling *Inference* Feeling Observation *Inference*

### Skills domains, ‘word pictures’ of students and ‘word summaries’

Five domains of working conceptualisations emerged in participants’ interviews:

1. Application of knowledge
2. Manner with patients
3. Getting it done
4. Safety
5. Overall impression

These are conceptually different from current discrete sequential or task-based domain categorisations of skills currently used in our medical school assessment rubrics (Lefroy et al. 2011). Instead participating assessors described working conceptualisations which were cross-cutting throughout the consultation. Three domains (those richest in data due to level of assessor attention paid to them namely: Manner with patients, Safety, and Overall impression) are discussed in more detail and illustrate the judgement mechanisms, ‘word pictures’ and ‘word summaries. Participants’ working conceptualisations described do not appear across all grades within each domain in the raw data (i.e. assessors made choices about what to apply and when) and analysis reflects this. Data extracts from participants are in double quotation marks (“) and extracts from the ‘word summary’ or ‘word picture’ are in single quotation marks (‘).

#### Manner with patients

Table 2 illustrates how the three judgement mechanisms (observed behaviours, inferences and feelings) emerged from discussion of students’ consultation skills judged over four grades from ‘fail’ to ‘very good’. For the domain ‘manner with patients’, examples of working conceptualisations identified in ‘word summaries’ for specific grades are presented below. ‘Word summaries’ were summarised from ‘word pictures’ which intentionally drew closely on participants’ natural language. Future stakeholders could draw on the ‘word picture’ to place and grade students on a scale if further clarification is needed to support their judgement.

For example, the ‘word summary’ judgement inferred by participants for a ‘borderline’ student’s manner with the patient was ‘Lacking in confidence, insufficient practice with patients’. The conceptualisation demonstrated in this ‘word summary’ ‘insufficient practice with patients’ drew on the ‘word picture’: ‘Some patients may be upset by what the student has said. Students not used to talking with patients, has not been practicing consultations. Tick box consultation’. This ‘word picture’ in turn developed from the raw data with supporting extracts: “Not had as much experience as they should, possibly upsetting a patient” (Assessor 9); “The patient doesn’t feel listened to and starts to switch off from the doctor; having forgotten what’s already been said” (Assessor 6); “little conversation, conversing only the clinical bit, focusing on the task” (Assessor 10).

In contrast, with a ‘very good’ student, participants ‘felt’ ‘reassured (about skills to work with patients)’. This conceptualisation emerged from the raw data and the ‘word picture’: ‘Reassurance that student knows what they are doing. Able to be human and warm as well as professional. Creates the beginnings of a doctor-patient relationship.’ This ‘word picture’ closely relates to data with exemplifying extracts that the student “Conveys a degree of reassurance that they know what they’re doing” (Assessor 1) and have the “beginnings of patient doctor relationship” (Assessor 9).

#### Safety

***‘***Safety’ was a prominent feature of participants’ discourse. Working conceptualisations of the ‘safety’ domain were underpinned by the three judgement mechanisms. Key conceptualisations identified in ‘word summaries’ drew on raw data from participants as described below (Table 3):

- Harm: Candidates who were observed to either physically or emotionally hurt or whose actions could harm the patient were flagged as potentially failing (Assessors 7, 10).
- Awareness: If participants inferred that candidates were unaware of the hurts and harms they caused or may have caused; the candidate was considered to be failing while those who exhibited awareness were considered to be borderline (Assessors 7, 9) and if students changed their approach to reduce hurt or harm they were considered to be of passing standard (Assessor 1).
- Potential for remediation: If participants inferred that students’ deficits were remediable, participants were likely to judge them borderline (Assessors 1, 7, 10).
- Trust: Any feelings of distrust (for example that student is ‘*worrying*’ (Assessors 7, 10), ‘*dangerous*’ (Assessors 2, 10), ‘*cannot be trusted*’ (Assessor 6) or ‘*scary on their own*’ (Assessor 3)) led to a fail. Conversely if the participant felt the student had demonstrated ‘*honesty in mistakes*’ (Assessor 10) this led to a borderline judgement.

#### Overall impression

‘Overall impression’ denotes a set of descriptions of ‘the impression the student made on me’ with which these participants informed their assessment. In these descriptions, participants’ judgements were more abstract, often based on inferences and feelings than descriptions of what students did (“Appendix 6”). Across different grades and judgement mechanisms, several key conceptualisations were identified in the ‘word summaries’ (Table 3) and are supported with data extracts below.

- Being a professional: with very good students participants described feeling like they are ‘*beginning to act and think like a doctor*’ (Assessor 9, 12): assessors feel happy to have them as a foundation doctor [intern] and feel “*you almost forget that they’re a medical student*” (Assessor 9).
- Managing emotions: participants inferred failing students may get so angry, upset or ‘‘*petulant*’’ they are unable to continue (Assessor 10), whereas borderline students may be perceived as ‘’*nervous*’’, or demonstrate ‘’*panic*’’ or ‘*’inappropriate emotion*’’ with some impact (Assessors 2, 4, 6) but are able to continue.
- Insight: with failing students, participants inferred they “*lack insight or don’t know they are wrong*” (Assessor 4).
- Taking responsibility for their actions: with a failing student participants may infer students are “*not accepting responsibility for own learning or for care of the patient*” (Assessor 4). Whereas a student who a participant inferred was “*conscientious*” (Assessor 12) was graded ‘*very good*’.
- Attitude: participants inferred that borderline students may have attitudinal problems: not taking the ‘*exam seriously or acting*’ (Assessor 3, 5, 12) or being: *overconfident or arrogant*” (Assessor 7).

### Grade descriptors

‘Grade descriptors’ encapsulate participants’ descriptions of students drawing on one or more of the three judgement mechanisms in each domain. They were developed from ‘word summaries’, ‘word pictures’ and the raw data for all five domains (Table 4). For example, in the knowledge domain, seemingly unthinking application of a routine untailored approach defines a failing student, whereas a passing student has a tailored approach. Some conceptualisations occurred only within one grade of one domain, for example, ‘able to rectify mistakes’ in the ‘pass’ grade of ‘safety’ (Table 4).

Across domains, ‘borderline’ grades were described using a mixture of ‘fail’ and ‘pass’ characteristics and being able to respond to feedback or improve.

Across domains, ‘very good’ grades were described as exceeding expectations and showing flexibility and adaptability to situations with some participants reflecting that a student’s consultation skills were better than his/hers at that stage.

## Discussion

The core of our findings describes assessors’ idiosyncratic reasoning thus highlighting the need to pay more attention to this in the design of assessment tools. Participating assessors used their working conceptualisations when forming exit standard consultation skills assessments based on three mechanisms of judgement (what they saw students do, inferences about the meaning of students’ actions, and how students made them feel) across four skills domains, ‘Application of Knowledge’, ‘Manner with patients’, ‘Getting it done’ and ‘Safety’ and one more abstract skills domain of ‘Overall impression’. While some of the domains identified correlate with those commonly present in rubrics generated using expert consensus, this study provides novel data on how these domains are operationalised in practice through working conceptualisations of assessors. Furthermore, expert consensus rubrics don’t address how assessors variably choose to draw on observation, interference and feelings in qualitatively evidencing their judgements and making choices about how to weigh these different mechanisms in different domains.

The five domains identified have some resonance with findings in postgraduate training assessment studies but do not match completely. Domains described for postgraduate assessment tend to be broader; for example, clinical skills and professional behaviour (Verhulst et al. 1986), task factors (what was done), humanistic factors and how the task was done (Lee et al. 2018) or think and act like a clinician (GP), the doctor-patient relationship, handling the biomedical aspects, and time management and structuring the consultation (Govaerts et al. 2013). Other studies have pointed towards a general impression being the only category in assessment of performance (Cook et al. 2010; Pulito et al. 2007) with a ‘halo’ effect present across rating domains (Govaerts et al. 2013). In their undergraduate work, Huntley et al. (2012) described two factors in their communications skills tool, the first concerning empathy and consulting style, the second around non-verbal aspects and professional behaviour, which was either scored as either competent or unacceptable, and may align with elements of safety and overall impression in our findings.

There are also some similarities with current research around how assessment judgments are made. Yeates et al. (2013) describe postgraduate assessors making emotive judgements such as ‘immediate dislike’ and global interpretive judgements such as ‘difficult to fault’. Others describe assessors making inferences (Gauthier et al. 2016; Gingerich et al. 2011, 2014; Novack et al. 1993; Rowntree 1987; Stillman et al. 1997). Inferences have been conceptualised as undesirable and contributing to the variability of assessment particularly when they are unverified (Kogan et al. 2011). A contrasting perspective is that inferences are part of a richer, context specific analysis of the situation (Gingerich et al. 2011, 2014; Govaerts et al. 2011, 2013). Similarly, assessors’ feelings have been shown to contribute to decision making (Gingerich et al. 2014). Such impression-making is part of knowing another person and is a synthesis of factual information, inferences, and evaluative reactions regarding the person (Hamilton et al. 1989). While this was largely postgraduate assessment research our data demonstrate similar judgements amongst undergraduate assessors. Gauthier et al. (2016) have published a narrative review to synthesise the mechanisms assessors use when rating learners (Gauthier et al. 2016). What we call ‘Inference judgements’ might be compared to Gauthier et al’s ‘Observation phase’ described by (‘Formulating high-level inferences’). What we call ‘Feelings’ could align with ‘Generating automatic impressions about the person’ but they have discounted ‘Feelings’ as a mechanism although they used have accessed overlapping literature (Gingerich et al. 2014). What we call ‘observation of behaviours’ is partly covered by Gauthier et al’s ‘Focusing on different dimensions of competencies. However, most of the studies in their synthesis were from the context of workplace based assessment and they describe assessors as only directly observing knowledge and clinical reasoning skills and using the learners’ case presentations to infer history taking and examination skills. Our participants have therefore provided a more granular description of such mechanisms in their judgements about consultation competencies in the context of OSCE assessment.

The ‘overall impression’ domain was most challenging to synthesise into ‘grade descriptors’. Participants described inferences and emotional responses more often than observed behaviours, and five key disparate conceptualisations were identified. This may be because assessors hold different values in relation to the ‘standard of being ready to enter the first year of training as a doctor [intern] (exit standard). Or it may be the data is evidence of assessors applying stereotypes or ‘person models’ (Gingerich et al. 2011) i.e. basing their judgments on the type of person they perceive to be in front of them, not the behaviours the person is displaying during the assessment and, consequently, it is difficult for assessors to describe the behaviours on which they are basing overall judgements.

We note that ‘safety’ was strongly present in our data. It is debatable whether this is a ‘product of the times’ that has pervaded undergraduate assessment from the contemporary wider clinical and political focus on safety (Francis 2013) or indicates assessors’ sense of responsibility for permitting students to ‘join their profession’ or an alternative explanation exists. Social judgements of morality have been related to judgements made in assessments, highlighting that humans can use dichotomised scales of competence/incompetence versus moral/immoral to make judgements (Gingerich et al. 2011; Wojciszke 1994). These dichotomised judgements share some conceptualisations with our participants’ descriptions of ‘safety’. Judgements that students were either incompetent or immoral were described in the fail grade of ‘safety’. However, ‘safety’ is a complex conceptualisation, particularly when considering the differing responsibilities and learning needs of medical trainees before and after becoming doctors.

Grades within each domain are not uniformly populated with working conceptualisations. An inference that a student is ‘judgemental’ about patients may place the student in the ‘fail’ category, but absence of a ‘judgemental’ inference does not appear in the ‘pass’ category whereas the inference that a student is ‘empathetic’ does. They may be two ends of a spectrum, dichotomised working conceptualisations (Gingerich et al. 2011) or representations of separate working conceptualisations.

### Strengths and limitations

Strengths of the study include that all authors dually work as clinicians and research methodologists who trained at and subsequently taught at different institutions. Our methodology was informed by previous empirical and theoretical work (Blumer 1969; Borman 1987; Crossley et al. 2002; Crotty 1998; Gingerich et al. 2011; Goffman 1967; Govaerts et al. 2013; Lefroy et al. 2011). We employed multiple techniques to ensure rigor and trustworthiness in both data generation and analysis and continued data generation until theoretical saturation was reached. Asking participants to give examples of practice and justify their explanations allowed us to generate data that could be analysed for mechanisms of applied practice, taking a critical stance. In this way we have gone beyond considering what assessors purport to do in the abstract (as would be generated in a standard setting exercise) to seeking how this translates into their working conceptualisation and applied thinking. We repeatedly cross-checked and critiqued each other’s interpretations. While we acknowledge that we have not addressed between-assessor differences in language in this study, it was not designed to do so but instead looked for commonality and we accept a different study might valuably look at differences. We believe this is the first study of its kind in an undergraduate setting and replication and further studies in more than one institution and across different forms of assessment are needed.

A study limitation is that interviews were structured using skills categories drawn from the institution’s assessment scale to ensure all aspects of the consultation were explored. This may have impacted on how participants reported their working assessment scales, and also interpretation of the domains and ‘word summaries’. To mitigate this impact, each domain was critiqued by an author without close knowledge of the local assessment scale. Several techniques were used in interviews to ensure participants’ descriptions of their judgement processes were as close to their actual practice and with as little priming as possible (Teunissen et al. 2009): participants were asked to start with a blank sheet, challenged if they used jargon and asked to draw on specific examples from their own practice. It is striking however that, apart from the four-category scale, the key findings of three different types of judgement and the domains which emerged are different from the local assessments.

We acknowledge the limitation that participants were asked to explain their actions and justify these when verbalising thoughts (Govaerts et al. 2013). Their accounts may not reflect their actual judgement processes which are often automatic, unintentional judgements (Bargh and Chartrand 1999) and may be post hoc rationalisations. However, given one cannot directly observe another’s thinking, our interviews were designed to minimise this effect and inferences and feelings described in this study suggest that we were able to gather some participants’ unintentional judgements which had not been rationalised in this way. Clinical assessors may be unwilling to describe healthcare trainees as having ‘failed’ (Dudek et al. 2005; Donaldson and Gray 2012). The extent to which the anchor point ‘clear fail’ may have affected participants’ reported judgements of failing students is uncertain.

We considered that member-checking (i.e. returning the analysis to participants) was not appropriate in this study. Some qualitative methodologists may disagree with this although limitations of member checking have been described (Mays and Pope 2000; Thorne 2017). Our rationale was that firstly, final outcomes are two stages of interpretative analysis from raw data and no longer have a direct relationship to individual participants’ working views. Secondly, final outcomes result from synthesis of multiple respondents’ source data. A single respondent may recognise aspects of their own contributions but not those of others. Finally, there is evidence that individual assessors weigh aspects differently depending on the individual and the task (Govaerts et al. 2013; Kogan et al. 2011). This study does not capture variation in how participants weighed different aspects of domains. A balance was intentionally drawn between being inclusive of participants’ different perspectives and conceptualisations and aiming for consensus and best representation of key conceptualisations relevant to most participants.

If one was to take a purely cognitive view on assessment it might be questioned whether our findings are a product of poor assessor training. As indicated in our study rationale and data, examiners do not take a purely cognitive approach to assessment and this is regardless of training. All our participants were experienced examiners who had engaged with the training requirements of the medical school, and these were comparable to training requirements commonly used as best practice elsewhere.

Finally, the assessment rubric is of necessity brief: it needs to be a document which is usable by assessors undertaking a cognitively challenging task (Tavares and Eva 2013, 2014). However, we do not intend that the rubric is used on its own but is ‘underpinned’ by the word summaries and pictures which should be freely available to all stakeholders in the assessment process.

## Implications for practice and research

We have shown that it is in the application of judgement that working conceptualisations come to the fore of assessors thinking and hence training in knowledge of assessment rubrics may always be ‘trumped’ by assessors’ prior experience and intuition when interacting with students. Our data suggests assessors who deviate from standard rubrics may be doing so in the belief that overlooked significant factors are at play, rather than because they do not understand how to apply the rubric consistently.

That working conceptualisations are identifiable is an exciting finding and encouraging for this field of research. Remaining questions include: are these conceptualisations shared by a larger, multi-institutional cohort of assessors within different contexts? Could assessment tools using working conceptualisations and natural language descriptors reduce the potential error in translation between assessors’ conceptualisations and an external rubric (Gingerich et al. 2011). In addition, utility of ‘word pictures’, ‘word summaries’ and ‘grade descriptors’ in assessment and training requires further investigation. For example, would ‘grade descriptors’ aligned to working conceptualisations and avoiding the word grade ‘fail’ reduce assessors’ reluctance to fail seen in other contexts (Donaldson and Gray 2012; Dudek et al. 2005)?

Assessment tools aligned to clinician assessors’ working conceptualisations may help students understand for example, professional concerns around safety, the need to respond constructively to errors, the mismatch between checklist and global scores (Hodges and McIlroy 2003) and the importance of spending time with patients and developing fluency of practice to ‘look like a doctor’. Challenges include how resulting assessments can be communicated to students in a ‘comprehensible and usable form’ and the defensibility of assessment decisions based on nominal data from such categorical sources (Gingerich et al. 2011).

## Conclusions

Our findings demonstrate that experienced clinicians use identifiable working conceptualisations when assessing undergraduate medical students’ consultation skills. We have also demonstrated that assessment tools drawing on participants’ conceptualisations and natural language can be generated, including ‘grade descriptors’ for common conceptualisations in each domain by mechanism and matched to the commonly used grading rubric of Fail, Borderline, Pass, Very good. These tools are aligned to the ‘real life’ approach taken by clinicians in assessing undergraduate consultations skills. Further work is needed to explore application of the research findings including prospective utility for assessors and institutions, and the impact on assessment quality.

## Figures and Tables

**Fig. 1:**
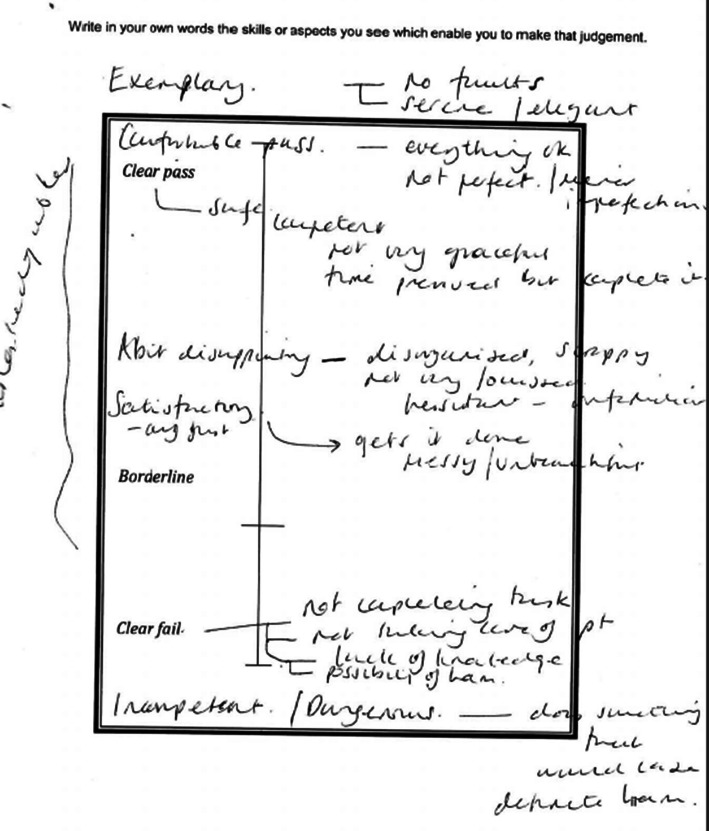
Example of reference points on a scale, annotated by assessor 11 for the ‘overall impression’ domain

## Appendix 1: Example of scales using during interviews

See Fig. 1.

## Appendix 3: Summary of stages of research

**Table:**

Stages of Research	Process	Outcomes and examples
Pilot interviews	5 performed by 2 interviewers, sharing notes, then standardizing first formal interview	Development of blank scale (Fig. 1), topic guide for interviews and initial coding framework (“Appendix 1, 2”)
*Primary analysis within and across interviews: developing domains*		
Initial interviews with assessors annotating scales	All interviews performed by same two interviewers. Interviewers transcribed talk around judgments into an initial coding framework. Critique of coding by second researcher	Initial coding framework refined (“Appendix 4”)
Round table meeting	Discussion of interview data, presented in interviewer-critiquing researcher pairs categories and emerging concepts	Additional category emerged—safety
Further interviews and round table meetings	Initial categories of skills, emerging concepts and domains explored in interviews, and tested in meetings until data saturation	Development of provisional domains
	Data from all interviews combined and analyzed across the interviews. Participant quotations which fitted within a domain were recorded. Any quotations and concepts which did not fit were highlighted	Domains populated with data across all interviews (See Table 2 for examples of assessors quotes)
Round table meeting	Discussion of the analysis, and challenging quotations and concepts. Discussion of how to make sense of types of judgments and distil quotations	The analytic framework was refined to include types of judgments made by assessors: observation, inferring and feeling
*Secondary analysis across domains: developing ‘grade descriptors’*		
*Stage 1* Development of word pictures—a description using assessor’s’ language and concepts which could be used to grade a student	The word pictures for each domain synthesized by one researcher, then critically reviewed by a second	Word pictures developed (see Table 2 for an example)
Round table meeting	Word pictures were discussed and critiqued. Consensus that further analysis was possible, to identify key concepts for each type of judgment, and descriptions of each grade	
*Stage 2 and 3* Development of word summaries—distilling key concepts for each type of judgment and grade descriptors—identifying the concepts of each grade	The word summaries and grade descriptors for each domain synthesized by one researcher, then critically reviewed by a second	‘Word summaries’ (see Table 4) and ‘grade descriptors’ agreed (see Table 5)
Round table meeting	Discussion and agreement of word summaries and grade descriptors	
	At each stage of the analysis we checked back to the previous stage and the original data to ensure consistency with the language used by assessors. This ensured the natural language was used to create the products of our analysis and drew on it in generating the descriptors	

## Appendix 5: Demographics of assessors. To preserve anonymity, participants are listed in order of years of experience as teachers rather than in the order in which they were recruited

**Table:**

Medical role	Gender	Age	Number of years involved in teaching	Number of OSCEs assessed	Number of workplace-based assessments completed
Emergency medicine	F	50	4	10–20	10–20
Elderly medicine	M	39	5	10–20	0
General practitioner	F	51	5	10–20	0
Elderly medicine	M	51	6	10–20	10–20
Surgeon	M	53	10	10–20	5–10
Neonatologist	F	50	10	20–30	0
Anaesthetist	F	56	11	> 30	10–20
General physician	M	43	12	10–20	0
Obstetrician and gynaecologist	F	50	20	> 30	100
Paediatrician	M	48	20	10–20	10–20
Gastroenterologist	F	53	29	10–20	0
General physician	F	45	22	10–20	10–20

## Apppendix 6: Skill domain ‘Overall impression’ showing how assessors’ raw data, with illustrating extracts were synthesised into ‘word pictures’ and ‘word summaries’ for each type of judgement (what the student does, what I infer, what this makes me feel)

**Table:**

Judgement type	Fail	Borderline	Pass	Very Good
What the student does				
Example data extracts*	Using the ‘I’m here as a student’ excuse in response to examiner probing (8). He was lying; making up physical signs, making out you can find something (2). Not trying; not concerned if they can’t do the task (3) Inappropriate dress (12). Became petulant, hugely unprofessional and the simulated patient was looking very worried (10)	Treat the exam as pretend; has awareness—potential to change (3). Inappropriate dress (12)	Can handle patient questions when they themselves don’t know the answer; knows where to go next, how to find things (3). Keeps thinking and does not panic (6). Presents self well (8). Performs as taught (12)	No unnecessary repetition (12). Look less anxious (11). Good students have the demeanor (6). Appears to be listening; checks understanding; completely thorough; makes the right judgement (6)
Word picture	Inappropriate dress, dishonesty or not caring for the patient. The simulated patient reports concerns about the student. Not performing as has been taught. Does not recognise or adjust behavior during exam or respond to feedback by examiner	Inappropriate in a minor way with regards to dress, skills, attitude or behaviour. May adjust behaviour during exam or recognise the problem during questioning	Performs as taught. Appropriate dress honesty and care of the patient, in line with training	Performs better than expected. Appropriate dress, honesty and care of the patient. Able to perform tasks completely and thoroughly and reach reasonable conclusions
Word summary	Inappropriate dress, dishonesty or not caring for the patient. Does not recognise failure or respond to feedback	Inappropriate in a minor way with regards to dress, skills, attitude or behavior. Recognises failure or responds to feedback	Appropriate dress, honesty and care of the patient, in line with training	Exceeds expectations
What I infer				
Example data extracts*	Resistance to conformity (12). Truly unhappy (3). Can’t be supported; no attitude of hard work; not coming across as taking responsibility for learning; or for good medical practice; not being responsible; uncompromising; lack of insight/don’t know they are wrong; wrong attitude (4). Unresponsive (to prompts); fails to demonstrate what they were taught (e.g. patient identification) (12). Never going to get there: became petulant; not completing the task; hugely unprofessional (10)	Treat the exam as pretend (3). Unconvinced of extrapolation to real life (12). The impression is that they are only trying because it’s an OSCE, it doesn’t seem that they are always like this (5). Inappropriate emotion or attitude; wrong attitude mixed with less than perfect knowledge (4). Demonstrating insight and ability to remediate for self (10). Needs support (12). Errs confidently, over-confident (7). Slightly panic that they’ve got to get it all done (2). Visibly nervous (6)	Coherent (3). Good defined as exam technique as well as skills to become a clinical scientist (5). Not arrogant (11). I can see they are competent even though they have made mistakes (11). Meets the criteria given; follows professional codes; situational awareness; recognising when the consultation is not going as expected (12), understands why they are doing what they are doing (12, 3)	[Perform] as on a post take ward round like a foundation year doctor (9). Absolutely brilliant, perfect, better than postgraduate student (11). Being in control of themselves; being comfortable enough to see the whole picture which includes the patient’s perspective (10). Compassionate professional and team competencies (7). Conscientious; Appropriate responding; Not over-confident; Working at the level of an F1 (12). Slick (6)
Word picture	Not accepting responsibility for their own learning or for care of the patient. Careless, uncompassionate, not in control of themselves or the situation. The patient is concerned about the student. Lacks insight into problems. Does not want or could not be supported to improve	The student is not taking the exam seriously or is acting. Lacking knowledge and skills expected. Has some insight into problems. Needs and can be supported to improve. Attitudinal problems overconfident or arrogant or too nervous to perform	Follows professional codes and meets the criteria given. They are competent, able to recognise mistakes and challenges in the consultation and respond to these. Generally, manages emotions—not panicking	Conscientious, compassionate, in control of themselves and the situation. Performs as a Foundation doctor or exceeds this or their level of training. Accepting responsibility of own learning and care of the patient
Word summary	Does not accept responsibility for own learning or for care of the patient. Uncaring. Lacks insight	Does not accept enough responsibility in this situation. Not caring enough or other attitudinal issue is present. Has some insight	Accepts responsibility in this situation. Recognises and responds to mistakes in real time. Has insight	Capably accepts responsibility in this situation. Conscientious, compassionate and in control of self and situation
What this makes me feel				
Data extracts	Bottom-feeders; unacceptable (3). Wouldn’t be happy to have as junior doctors; bad, erroneous judgement (1)	Expected basics; practical patient management (7). Will be okay (10)	Happy this person is going to be the house officer (1.) Just good enough (7)	Exemplary- as perfectly as I would want them too, there was nothing wrong (10). Everything ok, minor imperfections (7). Happy to have as junior doctors; you almost forget that they’re a medical student (9)
Word picture	I am concerned about the student having contact with patients or progressing further in the course	There are issues that student will work and can be supported to improve. The sense that exam situation is significantly impacting on the students’ performance	I am happy for the student to have contact with patients. Beginning to think and act like a doctor	I am happy for the student to work with patients. They are acting like a doctor, make you forget they are a student. I would want to work with them
Word summary	I am concerned about the student having contact with patients or progressing further in the course	The student will work on professionalism issues discovered, can be supported to improve. Exam impacts significantly	I am happy for student to have patient contact. Beginning to think and act like a doctor	I am happy for the student to work with patients. Performs like a doctor. I would want to work with them

*Note example data extracts only are shown for some grades due to space limitation. (Full tables can be requested from the corresponding author
